# Interspecies Scaling of Antibody–Drug Conjugates (ADC) for the Prediction of Human Clearance

**DOI:** 10.3390/antib10010001

**Published:** 2021-01-07

**Authors:** Iftekhar Mahmood

**Affiliations:** Mahmood Clinical Pharmacology Consultancy, LLC, Rockville, MD 20850, USA; Iftekharmahmood@aol.com; Tel.: +1-301-838-4555

**Keywords:** ADCs, allometric scaling, single and multiple-species scaling, pharmacokinetics, clearance

## Abstract

Allometric scaling is a useful tool for the extrapolation of pharmacokinetic parameters from animals to humans. The objective of this study was to predict human clearance of antibody–drug conjugates (ADC) allometrically from one to three animal species and compare the predicted human clearance with the observed human clearance. For three animal species allometric scaling, the “Rule of Exponents” (ROE) was used. The results of the study indicated that three-species allometric scaling in association with the ROE provides acceptable prediction (within 0.5–2-fold prediction error) of human clearance. The two-species allometric scaling resulted in substantial prediction error. One-species scaling using a fixed exponent of 1.0 provided acceptable prediction error (within 0.5–2-fold) by monkey, rat, and mouse, in which monkey and rat were comparable. Overall, the predicted human clearance values of ADCs from animal(s) was good. The allometric method proposed in this article can be used to predict human clearance from the animal data and subsequently to select the first-in-human dose of ADCs.

## 1. Introduction

Antibody–drug conjugates (ADCs) are therapeutic products consisting of an antibody linked to a biologically active cytotoxic drug forming a conjugate. Initially, most of the ADCs were developed for the treatment of oncology and hematology, but now, attempts are being made to expand the indications to other diseases such as inflammatory diseases, atherosclerosis, and bacteremia [1,2]. The first ADC Gemtuzumab ozogamicin (trade name: Mylotarg) for the treatment of CD33-positive acute myelogenous leukemia was approved by the USA Food and Drug Administration (FDA) in 2000 [2]. Since then, there has been an enormous focus on developing ADCs by the pharmaceutical companies. Eight ADCs are currently approved by the FDA for clinical use [2].

Interspecies allometric scaling is widely used to predict human pharmacokinetic (PK) parameters from animal data [3]. This approach is useful for both small and large molecules. Considering that the clearance of a drug is the most important PK parameter, a lot of emphasis has been given to predict human clearance for drugs from animal data with as much accuracy as possible [3]. A lot of information on the interspecies scaling of small as well as large molecules (therapeutic proteins that includes monoclonal antibodies and non-antibodies) exist [4,5,6,7,8]. However, there are not many studies available related to the interspecies scaling of ADCs, and this is mainly because preclinical ADCs’ data are not readily available. 

Li et al. [9] attempted to predict the human clearance of ADCs from preclinical data. The authors used a multiple-species (two-animal species scaling for six ADCs and three-animal species scaling for two ADCs for a total number of eight ADCs) allometric scaling using the “Rule of Exponents” (ROE). The authors also used a single-species (monkey) scaling using a fixed exponent (1.0) to predict ADC clearance in human. Based on their analysis, the authors concluded that the monkey alone provided better results than the multiple-species scaling. 

The ROE is based on three methods and was introduced by Mahmood and Balian to predict the human clearances from animals [3,10]. At least three animal species are required to apply ROE correctly. Initially, the ROE was developed for small molecules, but later, Mahmood expanded its application to therapeutic proteins (monoclonal antibodies and non-antibodies) [4,5]. The application of ROE is based on the exponents of simple allometry. If the exponent of allometry is between 0.56 and 0.70, the simple allometry is considered a suitable approach for the prediction of human clearance from animal data. If the exponents of allometry are between 0.71 and 0.99 or ≥1.0 but ≤1.3, then the maximum life-span potential (MLP) or brain weight is considered a suitable approach, respectively. In the case of macromolecules, Mahmood noted that [4,5] the application of MLP even if the exponents of simple allometry are between 0.71 and 0.99 would result in the substantial under-prediction of the human clearance.

In their two-species allometric scaling, the authors ignored the underlying conditions for the application of the ROE. It has been mentioned by Mahmood [5] and Goteti et al. [11] that one should be very careful for the ROE application when only two animal species data are available. The clearance prediction with two-species scaling and the application of ROE can lead to substantial prediction error. This was noted for both small molecules and antibodies [5,11]. Furthermore, it was found [5] that the maximum life-span potential (MLP) is not applicable to antibodies even if the exponents of the allometry are between 0.71 and 0.99 [5]. In their manuscript, Li et al. [9] incorrectly mentioned that the MLP should be used when the exponents of simple allometry were <0.71 (this may be a typographical error). In fact, MLP should be applied when the exponents of simple allometry are from 0.71 to 0.99 for small molecules but not for the therapeutic proteins.

Since the development of ADCs in the pharmaceutical industry is gaining momentum, it is necessary that the allometric scaling of ADCs be performed correctly. Therefore, the objectives of this study were as follows:

- To apply the ROE only to those drugs that had at least three animal species.
- Considering a real life situation where three animal species may not be available, two-animal species allometric scaling was performed to evaluate if it was possible to predict human clearance of ADCs with two-species allometric scaling using only simple allometry.
- The authors Li et al. used a single species scaling for the prediction of human clearance of ADCs. The species used by the authors was monkey. Although it is widely believed that the predicted human clearance can be fairly accurate using the monkey clearance values alone, there is no analysis with other species such as rat and mice for ADCs. Therefore, in this study, the clearance data from mice or rat were used to evaluate if the predicted human clearance values of ADCs are comparable with the monkey.
- Li et al. [9] used three allometric exponents (0.75, 0.85, and 1.0) for a single-species scaling for monkeys. The exponent 0.75 is a theoretical allometric exponent, exponent 0.85 was taken from Deng et al. [6,7] for the prediction of human clearance from monkey data, and the authors explored exponent 1.0. A study by Oitate et al. [8] indicated that the exponent 0.79 was the most suitable exponent for the prediction of human clearance of antibodies from monkey. Therefore, in this study, exponent 0.79 was also used for a single-species scaling by rounding it to 0.80.

## 2. Methods

A literature search was conducted to find some more PK data for ADCs than reported by Li et al. The search resulted in obtaining rat clearance data for DNIB0600A [12] and Polatuzumab vedotin [13], and mouse clearance data for brentuximab vedotin [14]. The PK data for thiomab and the anti-5T4 Antibody–Drug Conjugate was found in the literature with three animal species and humans [15,16,17,18,19]. Overall, there were seven ADCs for which three animal species and human clearance values were available for the scaling (Table 1). For seven ADCs, the total antibody clearance was predicted in humans and data for three ADCs (T-DM1, thiomab, and anti-5T4) were also available for the prediction of conjugated antibodies (total number of human clearance prediction was 10). The following allometric methods were used to predict human ADCs’ clearance from preclinical data. The ROE [10] was only used when three animal species were available for the scaling.

The following three methods were used (collectively known as ROE) to predict human clearance of ADCs from animal data.

### 2.1. Simple Allometry

The clearance was predicted by the following equation.

CL = a × (W)^b^ (1)

where ‘a’ is the coefficient and ‘b’ is exponent of the allometry. ‘W’ is the body weight. The weights of the species used in the scaling was 0.02, 0.25, 3.5, and 70 kg for mouse, rat, cynomolgus monkey, and humans, respectively (same body weight as used by Li et al.). The allometric scaling was done on an excel worksheet using the power function.

### 2.2. Product of MLP and Clearance (MLP × Clearance)

For mouse, rat, monkey, and human, the MLP values used in the analysis were 3, 4.4, 18, and 93.4 years, respectively [3,10].

In this analysis, the clearance of the species was multiplied by the MLP, and the product of the two was plotted against body weight. The human clearance of ADCs was predicted by the following equation.

CL × MLP = (a × (W)^b^)/93.4 (2)

where 93.4 (in years) is the human MLP.

### 2.3. Product of Brain Weight and Clearance (Br WT × Clearance)

For mouse, rat, monkey, and human, the brain weight used in the analysis was 0.36, 1.8, 63, and 1400 g, respectively. These brain weight values were used by Li et al. in their analysis.

In this analysis, the clearance of the species was multiplied by brain weight, and the product of the two was plotted against body weight. The clearance of ADCs was predicted by the following equation.

CL × Brain weight = (a × (W)^b^)/1400 (3)

where 1400 g is the human brain weight.

### 2.4. Two-Species Scaling

In this analysis, mouse and monkey, mouse and rat, and rat and monkey clearance values were used. The ROE is not applicable to two-species scaling and was not used.

### 2.5. One-Species Scaling

In this analysis, mouse, rat, or monkey clearance values were used for the prediction of human clearance of ADCs. The following equation was used for the analysis.

CL = Clearance in the species × (W)^b^ (4)

where ‘b’ is the exponent, and four exponents were used in the analysis (0.75, 0.80, 0.85, and 1.0). 

It was noted that when exponent 1.0 was used for mouse, the predicted human clearance was underestimated (less than the observed value) for few ADCs. In order to improve the prediction of ADC clearance in humans from mouse data, a different strategy was taken. In the first step, an exponent of 1.1 was used, but this exponent over-predicted the human clearance for some ADCs. In the second step, the clearance of ADCs in humans was predicted from three exponents (0.85, 1.0, and 1.1), and an average was taken and then compared with the observed human ADC clearance. This method was also used for rat and monkey. 

### 2.6. Statistical Analysis

The ratio between predicted and observed values were determined by the following equation.

Prediction Ratio = Predicted/Observed(5)

A predicted ratio between 0.5 and 2-fold (2-fold) error was considered acceptable. Although the percent error was not reported, but percent error is equal to (prediction ratio-1) × 100. For example, a prediction ratio of 1.3 means a 30% prediction error (higher than the observed clearance value).

## 3. Results

The clearance values for all ADCs in animals and humans are shown in Table 1 in absolute number rather than per kg body weight. 

### 3.1. Three-Species Allometric Scaling

There were seven ADCs that had three animal species (total number of observation 10 (seven for total antibody and three for conjugates). In Table 2, the coefficients and exponents as well as the predicted and observed human clearances of ADCs from three animal species scaling are shown. 

The exponents of allometry (10 observations) ranged from 0.82 to 1.07 (Table 2). The prediction/observed ratio for simple allometry ranged from 0.69 to 2.58. The prediction/observed ratio for simple allometry was within 0.5–2-fold for nine out of 10 observations (Table 2). The prediction error was >2-fold for only one ADC when simple allometry was used, indicating that simple allometry predicted the human clearance fairly well. 

For four ADCs, the exponents of simple allometry was >1, which indicated that brain weight as a correction factor was needed (Table 2). The incorporation of brain weight where needed in the scaling substantially improved the prediction of ADCs clearance in humans.

When brain weight as a correction factor was used, the prediction ratio ranged from 0.53 to 1.73 for all four ADCs. Overall, where needed, the combined use of simple allometry and brain weight improved the prediction of human clearance. Due to the small sample size, the true application and advantage of the ROE over simple allometry was not very clear. 

The clearance for three (T-DM1, thiomab, and anti-5T4) conjugated ADCs was also predicted in humans (Table 2). The exponents for all three conjugated ADCs were <1.0, and simple allometry predicted human clearance fairly accurately. The prediction error for T-DM1, thiomab, and anti-5T4 was 14%, 35%, and 9%, respectively. 

**Figure 1:**
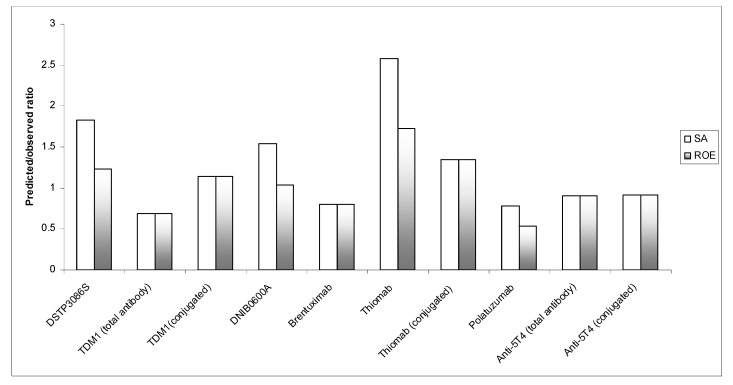
Predicted/observed ratio for the prediction of human clearance from three-species scaling using simple allometry (SA) and the “Rule of Exponent” (ROE). A predicted/observed ratio of 1 indicates no prediction error.

Overall, the results of the study indicated that a three-species allometric scaling can provide fairly accurate prediction of ADCs clearance in humans. For all 10 observations (total and conjugated), the predicted ratio was within 0.5–2-fold, nine of them being within 0.5–1.5-fold prediction error when the ROE strategy was used (Table 2 & Figure 1). 

### 3.2. Application of MLP:

There were six ADCs whose exponents (simple allometry) were <1 but >0.70, indicating that MLP could be applied (per small molecules). However, it was noted that the application of MLP to these six ADCs increased the prediction error, as the predicted values were substantially lower than the observed values (Table 3). This confirmed the previous observation of Mahmood [4,5] that MLP is not applicable to therapeutic proteins (monoclonal antibodies and non-antibodies), even if the exponents of allometry are >0.7 to 0.99. Hence, MLP is also not applicable to ADCs because it will lead to a substantial prediction error. 

### 3.3. Two-Species Allometric Scaling

The results of the two-species allometric scaling are shown in Table 4. There were 10 observations for mouse and monkey, seven observations for rat and monkey, and seven observations for mouse and rat. Simple allometry was used in this analysis, and no ROE was used in the allometric scaling. 

For mouse and monkey combination, the exponents of simple allometry were >1 for seven out of 10 observations. Seven out of 10 (70%) observations were within 2-fold prediction error (Table 4). 

For rat and monkey combination, the exponents of simple allometry were >1.0 for two out of seven observations. Four out of seven observations (57%) were within 2-fold prediction error (Table 4). There were two observations with >2-fold prediction error and one observation was <0.5-fold (Table 4).

The worst result was obtained for mouse and rat combination. Only three out of seven observations (43%) were within 2-fold prediction error (Table 4). 

Overall, the two-species scaling was unreliable and substantially erratic. A two-species allometric scaling for ADCs for the prediction of human clearance is not suitable and should not be used. Especially, the scaling from two rodent species (mouse and rat) provided the worst results. 

### 3.4. One-Species Allometric Scaling

The results of this analysis are shown in Table 5. Four fixed exponents (0.75, 0.80, 0.85, and 1.0) were used in this analysis. For monkey, the prediction ratio was within 2-fold for eight out of 10 (80%) ADCs by using exponent 1.0. The prediction error of <0.5-fold and >2-fold was for one ADC each (Table 5). When exponent 0.8 was used, there were eight out of 10 (80%) predicted human clearance values within 2-fold prediction error, whereas there were two ADCs with a prediction error of <0.5-fold. A similar result was noted with exponent 0.85. The worst result was with exponent 0.75 (four observations were under-predicted). Overall, exponents 0.80, 0.85, and 1.0 in terms of 2-fold prediction error provided similar results from monkey scaling. 

For rat, the prediction ratio was within 2-fold for five out of seven (71%) ADCs by using exponent 1.0. The prediction error was >2-fold for two ADCs. Using exponent 0.85, the prediction ratio was within 2-fold prediction error for five out of seven (71%) ADCS. The prediction error was <2-fold for 2 ADCs. 

For mouse, the prediction ratio was within 2-fold for six out of 10 (60%) ADCs. It was noted that in mouse, there were three ADCs with <0.5-fold and one ADC with >2-fold prediction error (Table 5). Overall, using exponent 1.0, best prediction was obtained from monkey, and the worst was obtained from mouse. 

**Figure 2:**
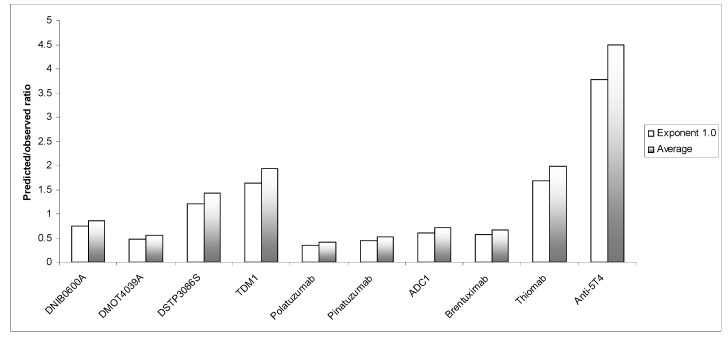
Predicted/observed ratio for the prediction of human clearance from mouse using exponent 1.0 or the average of different exponents (0.85, 1.0, and 1.1). A predicted/observed ratio of one indicates no prediction error.

In order to improve the prediction of ADCs clearance in humans from mouse, a different strategy was taken as described in the method section. Based on the use of multiple exponents, the prediction ratio was within 2-fold for eight out of 10 (80%) ADCs. The prediction error of <0.5 and >2 was for one ADC each (Table 5). The “average” strategy improved the prediction of human clearance of ADCs from mouse as compared with exponent 1.0 and was comparable with monkey (Table 6 & Figure 2). More data are needed to evaluate this method. If indeed, mouse clearance data can predict human clearance based on the proposed method (average of the exponents) as good as monkey, then this is a very useful approach. Cost-wise, mouse is much cheaper than monkey and also much easier to handle than monkey.

The multiple exponents did not improve the prediction of human clearance of ADCs for rat and monkey, since a single exponent was adequate enough to predict ADCs clearance in humans with reasonable accuracy. The prediction of ADCs clearance in humans from rat and monkey data using exponent 1.0 was approximately similar in terms of 2-fold error. 

## 4. Discussion

Considering the importance of ADCs in modern drug therapy, it is important that not only clinical but preclinical studies should also be conducted thoroughly. PK information from animal studies can be of practical value for not only understanding the absorption, distribution, metabolism, and elimination (ADME) processes for ADCs, but also, animal PK data can be used to estimate the first-in-human dose through interspecies allometric scaling. 

In their study, Li et al. incorrectly applied the ROE in their allometric scaling. This resulted in an incorrect estimate of human clearance for ADCs. The prediction of human clearance of drugs (small and large molecules) by three-species allometric scaling based on ROE will be in the following order: simple allometry > MLP > brain weight [10]. The ROE is a mathematical manipulation and has no physiological or biological meaning [10].

In two-species allometric scaling, this pattern may not be observed. For example, in the study of Li et al., the predicted human clearance was higher for brentuximab vedotin following the application of brain weight than simple allometry. Similarly, for other ADCs, the application of brain weight barely reduced (slightly >10%) the clearance values over simple allometry. On the other hand, the application of brain weight resulted in a >30% reduction in clearance over simple allometry when three animal species data were available (Li et al.’s study). The same observation was noted in this study (Table 2). Overall, the application of ROE is only appropriate when there are three or more animal species available. The ROE can also be applied to two animal species allometric scaling, but the accuracy may be compromised, and in many cases, one may get even worse results than the simple allometry [5,11].

Overall, the results of the study indicated that a three-animal species allometric scaling in association with the ROE provided human clearance with <2-fold prediction error (Table 2). However, this was not the case with two-species allometric scaling. The predicted human clearance by two-species scaling was substantially erratic, as discussed in the result section.

Although data were available for only three ADCs for the prediction of human clearance of conjugated ADCs, the results indicated that similar to total antibody, the human clearance of conjugated antibody could also be predicted as accurately as total antibody for ADCs.

In this study, in order to predict human clearance, different approaches were taken. In addition to using three or two-animal species scaling, a single-species scaling with a fixed exponent was also conducted. It is widely believed that using monkey and a single exponent, a similar result could be obtained as three or two-animal species allometric scaling. However, different studies found different fixed exponents, which was considered most suitable by the authors [6,7,8,9]. In addition to monkey, two rodents (mouse and rat) are also widely used in preclinical studies. Without systematic study, it has been assumed that mouse or rat will provide inferior results than the monkey. Therefore, in this study, besides monkey, mouse or rat clearance data were used for a single-species allometric scaling to predict human clearance of ADCs.

For a single species scaling, when a fixed exponent 1.0 was used 80%, 71%, and 60% prediction error were within 0.5–2-fold for monkey, rat, and mouse, respectively. Since the exponents of allometry widely vary and are data dependent, a single exponent many not provide a desirable prediction error (within 0.5–2-fold prediction error). 

It should be recognized that the use of a single exponent to predict human clearance from a single species is unreliable, and it can vary from study to study. For example, Oitate et al. [8] and Deng et al. [6,7] found that exponent 0.79 or 0.85, respectively, was the most suitable exponent for the prediction of human clearance of antibodies from the monkeys. Li et al. found that exponent 1.0 was the most suitable exponent for the prediction of human clearance of ADCs from monkey. However, in this study, it was noted that the exponent 0.80 and 0.85 provides similar results as obtained from exponent 1.0. These studies indicate variability in deciding the best exponents for the prediction of human clearance from a single species for antibodies and ADCs. 

In order to improve the predictive performance of one-species scaling, three different exponents 0.80, 1.0, and 1.1 were applied to all three species, and then, the average was taken from the predicted human clearance from these three exponents. This approach did not improve the human clearance prediction from rat or monkey clearance data but it improved from mouse clearance data. As compared with a single exponent of 1.0, the use of multiple exponents improved the human clearance prediction from mouse clearance data by increasing the 2-fold prediction error from 60% to 80% and was comparable with monkey. A single exponent of 1.0 from mouse under-predicted the human clearance of ADCs but by using three different exponents, the under-prediction was reduced from three ADCs to one ADC. However, at this time, there are not enough data to evaluate the predictive power for the prediction of human clearance of ADCs from mouse or rat. However, the current analysis with mouse or rat is encouraging, and such a scaling should be conducted with more data, because this can be of practical value. It is also possible to improve the prediction of human clearance of ADCs from rat by taking the average based on exponents 0.85 and 1.0, and further investigation should be conducted in this direction. 

## 5. Conclusions

This study indicates that allometric scaling is a useful tool to predict human clearance of ADCs from animal data. A good result was obtained when clearance data from three species were available and the application of the ROE resulted with a prediction error within 2-fold for all seven ADCs. However, a two-species allometric scaling for the prediction of human clearance of ADCs is not suitable and should be avoided. Furthermore, the ROE should not be applied to two-species allometric scaling. In this study, it appears (although small data) that one-species scaling from mouse or rat or monkey can provide a similar result. Further work is needed to evaluate the predictive performance of one-species scaling, particularly in mouse or rat.

## Figures and Tables

**Table 1:** Body weight and clearance values for antibody–drug conjugates (ADCs) used in the analysis.

Drugs	CL (mL/day)	Drugs	CL (mL/day)
**DNIB0600A**		**Polatuzumab vedotin**	
Mouse	0.18	Mouse	0.11
Rat *	3.98	Rat *	4
Monkey	46.55	Monkey	24.2
Human	854	Human	1015
**DMOT4039A**		**Pinatuzumab vedotin**	
Mouse	0.19	Mouse	0.12
Monkey	96.6	Monkey	32.9
Human	1400	Human	966
**DSTP3086S**		**Brentuximab vedotin**	
Mouse	0.20	Mouse *	0.50
Rat	2.37	Rat	2.25
Monkey	46.9	Monkey	51.1
Human	574	Human	742
**T-DM1 (total antibody)**		**T-DM1 (conjugate)**	
Mouse	0.16	Mouse	0.38
Rat	1.62	Rat	4.6
Monkey	16.1	Monkey	41.9
Human	343	Human	600
**Thiomab (New) total antibody**		**Thiomab (New) conjugate**	
Mouse	0.10	Mouse	0.40
Rat	2.15	Rat	6.03
Monkey	20.37	Monkey	54.25
Human	200	Human	759
**Anti-5T4 (New) (total antibody)**		**Anti-5T4 (New) (conjugate)**	
Mouse	0.39	Mouse	0.68
Rat	3.3	Rat	4.8
Monkey	27.1	Monkey	52.6
Human	360	Human	700
**ADC1**			
Mouse	0.13		
Monkey	36.7		
Human	756		

* new species added to Li et al. data. Brain weight values in mouse, rat, monkey, and humans are 0.36, 1.8, 63, and 1400 g, respectively, as provided by Li et al. Body weight values in mouse, rat, monkey, and humans are 0.02, 0.25, 3.5, and 70 kg, respectively, as provided by Li et al.

**Table 2:** Predicted and observed clearances of ADCs by three animal species allometric scaling.

Drugs	Coefficient	Exponent	Observed	Predicted	Ratio *
**DSTP3086S (total antibody)**					
Simple	11.7	1.06	574	1052	1.83
Brain weight	155.7	2.06	574	709	1.23
**T-DM1 (total antibody)**					
Simple	5.37	0.89	343	236	0.69
**T-DM1 (conjugate)**					
Simple	14.3	0.91	600	683	1.14
**DNIB0600A (rat data were added to Li et al.’s original data) (total antibody)**					
Simple	13.7	1.07	854	1313	1.54
Brain weight	182.5	2.08	854	886	1.04
**Brentuximab vedotin (mouse data were added to Li et al.’s original data) (total antibody)**					
Simple	13	0.90	742	595	0.80
**Thiomab (total antibody)**					
Simple	6.5	1.03	200	517	2.58
Brain weight	86.9	2.03	200	345	1.73
**Thiomab (conjugate)**					
Simple	18.3	0.95	759	1027	1.35
**Polatuzumab vedotin (total antibody) (rat data were added to Li et al.’s original data) (total antibody)**					
Simple	8.91	1.06	1015	795	0.78
Brain weight	119	2.06	1015	537	0.53
Anti-5T4 (total antibody) **					
Simple	9.9	0.82	360	323	0.90
Anti-5T4 (conjugate) **					
Simple	17.3	0.85	700	640	0.91

* Predicted Ratio = Predicted/Observed. ** The human clearance of Anti-5T4 was based on a 4.34 mg/kg human dose for both total antibody and conjugate. ** The human clearance of Anti-5T4 was calculated by extracting the mean concentration–time data from Figure 2 in the original study.

**Table 3:** Predicted and observed clearances of ADCs by simple allometry (SA) and maximum life-span potential (MLP) using three animal species.

Drugs	Exponent	Observed	Predicted	Predicted
	3 species, SA		SA	MLP
**Brentuximab vedotin**				
Methods	0.90	742	595	274
Ratio			0.80	0.37
**T-DM1 (total antibody)**				
Methods	0.89	343	236	111
Ratio			0.69	0.32
**T-DM1 (conjugate)**				
Methods	0.91	600	683	319
Ratio			1.14	0.53
**Thiomab (conjugate)**				
Methods	0.95	759	1027	478
Ratio			1.35	0.63
**Anti-5T4 (total antibody)**				
Methods	0.82	360	323	151
Ratio			0.90	0.42
**Anti-5T4 (conjugate)**				
Methods	0.85	700	640	288
Ratio			0.91	0.41

**Table 4:** Prediction of human clearance of total antibody by two-species allometric scaling.

Drugs	Coefficient	Exponent	Predicted	Observed	Ratio *
**DNIB0600A**					
Mouse, monkey	12.1	1.08	1190	854	1.39
Mouse, rat	21.8	1.23	3981	854	4.66
Rat, monkey	14.5	0.93	753	854	0.88
**DMOT4039A**					
Mouse, monkey	21.3	1.21	3640	1400	2.60
**Polatuzumab vedotin**					
Mouse, monkey	6.6	1.04	543	1015	0.53
Mouse, rat	28.8	1.42	12,007	1015	11.83
Rat, monkey	10.3	0.68	185	1015	0.18
**Pinatuzumab vedotin**					
Mouse, monkey	8.46	1.08	832	966	0.86
**ADC1**					
Mouse, monkey	9.38	1.09	962	756	1.27
**Brentuximab vedotin**					
Mouse, monkey	16.6	0.89	728	742	0.98
Mouse, rat	5.1	0.59	63	742	0.08
Rat, monkey	11.6	1.18	1745	742	2.35
**DSTP3086S**					
Mouse, monkey	12.4	1.09	1277	574	2.22
Mouse, rat	9.3	0.98	598	574	1.04
Rat, monkey	11.4	1.13	1386	574	2.41
**T-DM1**					
Mouse, monkey	5.3	0.89	231	343	0.67
Mouse, rat	5.4	0.87	219	343	0.64
Rat, monkey	5.8	0.92	291	343	0.85
**Thiomab**					
Mouse, monkey	5.6	1.03	445	200	2.23
Mouse, rat	11.6	1.21	1982	200	9.91
Rat, monkey	7	0.85	259	200	1.30
**Anti-5T4**					
Mouse, monkey	9.7	0.82	316	360	0.88
Mouse, rat	10.7	0.85	388	360	1.08
Rat, monkey	10	0.80	299	360	0.83

* Predicted Ratio = Predicted/Observed.

**Table 5:** Predicted human clearance (total antibody) by different exponents from mouse, rat, and monkey.

Species	Predicted				Prediction Ratio			
	E 0.75	E 0.80	E 0.85	E 1.0	E 0.75	E 0.80	E 0.85	E 1.0
**DNIB0600A (observed human CL = 854 mL/day)**								
Mouse	82	123	185	630	0.10	0.14	0.22	0.74
Rat	272	361	479	1114	0.32	0.42	0.56	1.30
Monkey	440	511	594	931	0.52	0.60	0.70	1.09
**DMOT4039A (observed human CL = 1400 mL/day)**								
Mouse	86	130	196	665	0.06	0.09	0.14	0.48
Monkey	914	1061	1233	1932	0.65	0.76	0.88	1.38
**DSTP3086S (observed human CL = 574 mL/day)**								
Mouse	90	135	204	693	0.16	0.24	0.35	1.21
Rat	163	215	286	665	0.28	0.38	0.50	1.16
Monkey	444	515	598	938	0.77	0.90	1.04	1.63
**TDM1 (observed human CL = 343 mL/day)**								
Mouse	73	109	165	560	0.21	0.32	0.48	1.63
Rat	111	147	195	455	0.32	0.43	0.57	1.33
Monkey	152	177	205	322	0.44	0.52	0.60	0.94
**Polatuzumab vedotin (observed human CL = 1015 mL/day)**								
Mouse	46	70	105	356	0.05	0.07	0.10	0.35
Rat	185	245	325	756	0.18	0.24	0.32	0.74
Monkey	199	231	268	420	0.20	0.23	0.26	0.41
**Pinatuzumab vedotin (observed human CL = 966 mL/day)**								
Mouse	56	83	126	427	0.06	0.09	0.13	0.44
Monkey	311	361	420	658	0.32	0.37	0.43	0.68
**ADC1 (observed human CL = 756 mL/day)**								
Mouse	60	90	136	462	0.08	0.12	0.18	0.61
Monkey	348	404	469	735	0.46	0.53	0.62	0.97
**Brentuximab vedotin (observed human CL = 742 mL/day)**								
Mouse	55	82	123	420	0.07	0.11	0.17	0.57
Rat	154	204	271	630	0.21	0.28	0.36	0.85
Monkey	483	561	652	1022	0.65	0.76	0.88	1.38
**Thiomab (observed human CL = 200 mL/day)**								
Mouse	44	66	99	338	0.22	0.33	0.50	1.69
Rat	147	195	259	602	0.74	0.98	1.29	3.01
Monkey	193	224	260	407	0.96	1.12	1.30	2.04
**Anti-5T4 (observed human CL = 360 mL/day)**								
Mouse	177	266	400	1361	0.49	0.74	1.11	3.78
Rat	226	299	397	924	0.63	0.83	1.10	2.57
Monkey	256	297	345	541	0.71	0.83	0.96	1.50

**Table 6:** Predicted human clearance (total antibody) from mouse using two different methods.

ADCs	Observed CL	Predicted CL (mL/day)		Predicted Ratio	
	(mL/Day)	Exponent 1.0	Average *	Exponent 1.0	Average *
DNIB0600A	854	630	726	0.74	0.85
DMOT4039A	1400	665	766	0.48	0.55
DSTP3086S	574	693	799	1.21	1.43
TDM1	343	560	645	1.63	1.94
Polatuzumab	1015	356	411	0.35	0.42
Pinatuzumab	966	427	492	0.44	0.52
ADC1	756	462	532	0.61	0.72
Brentuximab	742	420	484	0.57	0.67
Thiomab	200	338	390	1.69	1.99
Anti-5T4	360	1361	1618	3.78	4.49

* The human clearance from mouse was predicted using exponents 0.85, 1.0, and 1.1, and then, average clearance values were used to predict final human clearance.

## Data Availability

Data are available from the references provided in this manuscript.
